# Characterization of the Oral Microbiome in Wearers of Fixed and Removable Implant or Non-Implant-Supported Prostheses in Healthy and Pathological Oral Conditions: A Narrative Review

**DOI:** 10.3390/microorganisms11041041

**Published:** 2023-04-16

**Authors:** Francesco D’Ambrosio, Biagio Santella, Maria Pia Di Palo, Francesco Giordano, Roberto Lo Giudice

**Affiliations:** 1 Department of Medicine, Surgery and Dentistry “Schola Medica Salernitana”, University of Salerno, Via S. Allende, 84081 Baronissi, Italy; 2 Department of Human Pathology in Adulthood and Childhood “G. Barresi”, University Hospital “G. Martino” of Messina, Via Consolare Valeria 1, 98123 Messina, Italy

**Keywords:** dental microbiome, oral microbiota, removable prostheses, fixed prostheses, teeth, dental implants, oral hygiene, oral microorganisms, dental plaque, oral biofilm

## Abstract

Oral commensal microorganisms perform very important functions such as contributing to the health of the host. However, the oral microbiota also plays an important role in the pathogenesis and development of various oral and systemic diseases. The oral microbiome may be characterized by a higher prevalence of some microorganisms than others in subjects with removable or fixed prostheses, depending on oral health conditions, the prosthetic materials used, and any pathological conditions brought about by inadequate prosthetic manufacturing or poor oral hygiene. Both biotic and abiotic surfaces of removable and fixed prostheses can be easily colonized by bacteria, fungi, and viruses, which can become potential pathogens. The oral hygiene of denture wearers is often inadequate, and this can promote oral dysbiosis and the switch of microorganisms from commensal to pathogens. In light of what emerged from this review, fixed and removable dental prostheses on teeth and on implants are subject to bacterial colonization and can contribute to the formation of bacterial plaque. It is of fundamental importance to carry out the daily hygiene procedures of prosthetic products, to design the prosthesis to facilitate the patient’s home oral hygiene practices, and to use products against plaque accumulation or capable of reducing oral dysbiosis to improve patients’ home oral practices. Therefore, this review primarily aimed to analyze the oral microbiome composition in fixed and removable implant or non-implant-supported prostheses wearers in healthy and pathological oral conditions. Secondly, this review aims to point out related periodontal self-care recommendations for oral dysbiosis prevention and periodontal health maintenance in fixed and removable implant or non-implant-supported prostheses wearers.

## 1. Introduction

The human being is a superorganism as it is made up of both its own cells and microorganisms that reside either on or inside the human body; the latter of which are ten times more numerous than the cells of the body [1,2]. Commensal microorganisms perform very important functions such as contributing to the health of the host, resisting pathogens, maintaining homeostasis, and modulating the immune system [3].

However, although the oral microbiota is of paramount importance for host health, it also plays an important role in the pathogenesis and development of various oral and systemic diseases [2].

In fact, under particular conditions, virulence factors of oral bacteria can reach distant organs or influence host immune responses [2,4]. Several systemic diseases have indeed been associated with conditions of oral dysbioses, such as chronic inflammatory bowel or degenerative diseases (e.g., atherosclerosis and Alzheimer’s disease), age-related macular degeneration, and cancers [4]. At the same time, the oral microbiota can be altered by systemic diseases such as diabetes [5].

On the other hand, an accumulation of bacteria and other microorganisms can occur and can induce the development of oral diseases. In fact, the most common oral diseases such as caries, gingivitis, or periodontitis are induced by microorganisms [6].

Oral microorganisms can form the oral biofilm, which is a three-dimensional structure with different communities of microorganisms immersed in an extracellular matrix [7]. Bacterial adhesion is preceded by the formation of an acquired film consisting mainly of salivary glycoproteins [7]. In the early stages, weak physicochemical interactions are formed between the different microorganisms and the acquired film. Later, stronger bonds are established between the bacterial adhesins and the glycoprotein receptors of the acquired film [7]. The microbial composition gradually increases due to the coaggregation of late colonizers that bind to the receptors of early bacteria [7].

Microorganisms’ adhesion can occur on both biotic and abiotic surfaces [8] such as removable and fixed prostheses that can be easily colonized by bacteria, fungi, and viruses, which can become potential pathogens.

Several studies have shown that after the complete loss of teeth, some of the species still remain in the oral cavity [9]. The oral microbiome may be characterized by a higher prevalence of some microorganisms than others in subjects with removable or fixed prostheses depending on oral health conditions, the prosthetic materials used, and any pathological conditions brought about by inadequate prosthetic manufacturing or poor oral hygiene. In addition, it has been highlighted how in the case of edentulous or non-dentulous prosthesis wearers [10] numerous bacterial species, present in the periodontal pockets and strongly associated with systemic pathologies, can be found in the mouth [11].

Removable implant or non-implant-supported and fixed prostheses on teeth or implants are more frequently found among elderly subjects. It should be emphasized that with advancing age, vision, and tactile sensitivity disorders increase, which leads to not noticing the biofilm on natural teeth, prostheses, and implant attachments [12]. Furthermore, degenerative diseases and the reduced manual dexterity of elderly patients often do not allow accurate removal of the biofilm that forms over time on natural teeth or any type of dentures, causing a significant increase in the bacterial load in the oral cavity [12].

The microbial change at the peri-implant level should be considered because these bacteria could then colonize the prosthetic artifacts. The new classification of periodontal and peri-implant diseases focuses attention on the circumstances that determine these conditions. In particular, the health of the implant is evaluated through the absence of clinical signs of inflammation, such as bleeding (BoP) and suppuration, a pocket depth (PB) in a physiological range, and the absence of peri-implant bone loss [13].

It has been described that the peri-implantitis microbiome is characterized by high values of aerobic Gram-positive, anaerobic Gram-negative, and spindle-shaped pathogenic rods [13].

Therefore, this review primarily aimed to analyze the microbiome composition of the soft and hard tissues of the oral cavity in fixed and removable implant or non-implant-supported prostheses wearers in healthy and pathological conditions. Secondly, this review aims to point out related periodontal self-care recommendations for oral dysbiosis prevention and periodontal health maintenance in fixed and removable implant or non-implant-supported prostheses wearers.

## 2. Materials and Methods

The main question is whether there are differences between the oral microbiome in fixed and removable implant wearers or non-implant-supported prosthesis wearers in healthy and pathological oral conditions, and what are the self-care recommendations for oral dysbiosis prevention and periodontal health maintenance in fixed and removable implant or non-implant-supported prostheses wearers.

An electronic search was conducted in PubMed, Scopus, Google Scholar, and Web of Science without date restriction until 26 February 2023 to search scientific articles describing the composition of the oral microbiome in healthy or pathological oral conditions in fixed and removable implant or non-implant-supported prostheses wearers.

An additional search was conducted for records describing oral hygiene measures that provide better control to avoid dangerous and undesirable oral dysbiosis in removable and fixed denture wearers.

The following keywords were used:

(Dental microbiome OR oral microbiota OR oral microorganisms OR dental plaque OR oral biofilm OR oral hygiene)

AND

(Teeth OR natural teeth OR dental implants OR dentures OR dental prostheses OR removable dentures OR removable prostheses OR fixed dentures OR fixed prostheses OR dentures).

References were exported and managed using Mendeley Reference Manager.

This review is a narrative review, so it is not based on statistical analyzes or bias reduction through confounding analysis.

## 3. Results

### 3.1. Oral Microbiome in Healthy and Pathological Oral Conditions

#### 3.1.1. Oral Microbiome in Healthy Oral Conditions

The oral cavity is characterized by different structures, corresponding to different ecological niches, such as the soft tissue surfaces of the oral and tongue mucosa, periodontal pockets, the surface of the hard tissues of the teeth, the implant surface, and saliva which constitute small microhabitats for numerous species [2,14,15].

In the oral cavity there is a temperature of about 37 °C and a salivary pH that oscillates between 6.5 and 7.5, which are ideal conditions for the growth of the oral microbiome; one of the more complex and diverse microbiomes of the human body. The oral microbiota is, in fact, characterized by more than 700 species and is one of the richest in species including bacteria, fungi, viruses, archaea, and protozoa [2].

There are six main phyla that characterize about 94% of the oral bacterial community and they are *Firmicutes*, *Bacteroidetes*, *Proteobacteria*, *Actinobacteria*, *Spirochaetes,* and *Fusobacteria*. The phyla, *Saccharibacteria*, *Synergistetes*, *SR1*, *Gracilibacteria*, *Chlamydia*, *Chloroflexi*, *Tenericutes,* and *Chlorobi*, make up only the remaining 6% of the bacteria (a detailed list of oral bacteria with a description of their specificities and genomic characteristics is available on the Human Oral Microbiome Database. Available at: http://www.homd.org accessed on 13 April 2023) [2,3,16,17].

In the oral cavity, the primary colonizers of hard surfaces are mainly Gram-positive *Streptococcus* spp. (*Streptococcus oralis*, *Streptococcus mutans*, *Streptococcus mitis*, *Streptococcus gordonii*, *Streptococcus sanguinis,* and *Streptococcus parasanguinis*) and other species including *Veillonella* spp., *Neisseria* spp., *Rothia* spp., *Abiotrophia* spp., *Gamella* spp. and *Granullicatella* spp., and only later would we find the secondary colonizers who would join and coaggregate with the primary colonizers [13]. This mechanism also occurs on prostheses, where obligate anaerobes are reported in the more mature plaque and therefore can be a marker to understand the maturation of the plaque and highlight poor hygiene of the prosthesis [18].

Viruses, although rare, can be found in the oral cavity and are often associated with disease. The viruses most frequently found in the oral cavity are the Herpes Simplex Virus (HSV) [19] and Human Papilloma Virus (HPV) [15]. In addition, blood-borne viruses, such as the Hepatitis Virus and Human Immunodeficiency Virus (HIV), can be found that can enter the mouth through the crevicular gum fluid [20]. Several Authors have recently described that SARS-CoV-2, which causes the COVID-19 disease, can be found in the oral cavity, highlighting how saliva could also be a vehicle of contagion [21].

Fungi are frequently present in the oral cavity, as they are part of the healthy oral microbiota, but in elderly and immunosuppressed subjects they can become opportunistic pathogens. The predominant genera present in the oral cavity are *Candida*, *Cladosporium*, *Aureobasidium*, *Saccharomycetales*, *Aspergillus*, *Fusarium* and *Cryptococcus* [2,3,16,17]. *Candida albicans* (*C. albicans*) is the most frequently encountered fungus in the oral cavity. In fact, *C. albicans* is an oral commensal present in almost 40–65% of healthy adults.

Archaea constitute a small part of the oral microbiome and consist of a few species including *Thermoplasmatales*, *Methanobrevibacter*, *Methanobacterium*, *Methanosarcina* and *Methanosphaera* [22,23]. They are found in healthy individuals, but their prevalence increases in individuals with periodontitis [23].

The protozoa most commonly found in the oral cavity are amoeba, *Entamoeba gingivalis,* and *Trichomonas tenax*. These protozoan species are found in the normal oral microbiome; however, in individuals with poor oral hygiene and gum disease, the species encountered may increase [24].

#### 3.1.2. Oral Microbiome in Pathological Oral Conditions

The oral microbiota present has an important role in maintaining oral and systemic health. In fact, the oral microbiota inhibits colonization by pathogens and the phenomenon of resistance to colonization [25]. Since all surfaces of the mouth are colonized by commensal organisms there are few binding sites available for pathogens.

This balance is well highlighted when the commensal microbiota is altered, for example by the use of antimicrobials and infections by opportunistic pathogens such as *Candida* spp. and *Staphylococcus aureus* [26,27,28].

Oral candidiasis often involves immunocompromised subjects [29] and can present in acute (pseudomembranous, atrophic or erythematous candidiasis, or angular cheilitis) or chronic form (atrophic meta-protective stomatitis, hypertrophic stomatitis, periodontal localization candidiasis, or HIV-associated erythematous) [30].

The viruses most frequently found in the oral cavity are the Herpes Simplex Virus (HSV) [19] which causes primary herpetic gingivostomatitis as the first manifestation of infection, affecting the oral and labial mucous membranes [31]; and herpes labialis on the lips and perioral skin as a manifestation of viral reactivation [32]; Human Papilloma Virus (HPV) which causes papillomas, condylomas, oral warts, and focal epithelial hyperplasia [15], and has also been implicated in squamous cell carcinoma of the head and neck [33,34]. The HIV virus can indirectly cause various oral manifestations, such as oral candidiasis, oral hairy leukoplakia, linear gingival erythema, necrotizing ulcerative periodontitis, and Kaposi’s sarcoma [20]. SARS-CoV-2 can directly or indirectly cause various primary lesions in the oral cavity such as erosions and ulcers, macules and petechiae, white or red plaques, vesicles and boils, and other manifestations [21,29,35,36,37].

### 3.2. How Can Prosthetic Materials Affect the Development of Oral Biofilm?

Many studies have evaluated the differences in the oral microbiome between subjects with fixed prosthetic restorations fabricated with different materials and manufacturing methods [38,39]. The results highlighted some peculiarities depending on the nature of the dental biomaterials and the production methods used.

The structure and chemical composition of the material of which the dental prosthesis is made should be considered; in fact, these can be important factors in favoring the colonization by microorganisms. The surface of the prosthesis is covered by an “acquired film” of salivary glycoproteins and immunoglobulins when inserted into the oral cavity just as occurs for the dental surface [40,41].

A study on polymethylmethacrylate (PMMA), the material usually used in the manufacture of dental prostheses, showed that the acquired films were mainly characterized by lyzozyme and histatin, unlike those present on the teeth and which were carbonic anhydrase, carbonate dehydrastasis, cystatin, and lyzozyme and histatins [42]. This coating and adhesive films facilitate the adhesion and colonization of microorganisms [43]. Another study described how microbial colonization is not necessarily given by the material that characterizes the prosthesis, with PMMA and polyacrylamide supporting biofilm growth [44].

The *Candida albicans* observed in the smear specimens resulted in an inflammatory reaction in the periodontium when using cobalt–chrome (Co–Cr)-based ceramic dental restorations of the conventional manufacturing technique, which was probably related to the weakened immunity in the gingival sulcus. It is worth mentioning that *Candida albicans* was found during bacteriological and bacterioscopic analyses of subjects with fixed prosthetic restorations, within 1 year of follow-up, which reliably confirmed their existence.

The same picture emerged when fixed Co–Cr ceramic restorations were fabricated using the Computer Aided Design/Computer Aided Manufacture (CAD/CAM) technique; in fact, there was a difference in the number of microflorae in subjects who received conventionally manufactured Co–Cr-based ceramic dental restorations.

Zirconium-based ceramic restorations showed the best results in the gingival sulcus qualitative and quantitative composition of the microbiota.

After 12 months of prosthetic rehabilitation with fixed restorations in (Co–Cr) ceramic and full coverage zirconium ceramic, a quantitative reduction of various microorganisms, such as *Prevotella intermedia*, *Streptococcus haemolyticus*, *Porphyromonas gingivalis*, *Fusobacterium* spp., and *Corynebacterium anaerobium* [45], were found.

### 3.3. Oral Microbiome in Fixed Dentures on Teeth in Healthy and Pathological Conditions

#### 3.3.1. Oral Microbiome in Fixed Dentures on Teeth in Healthy Conditions

According to a study by Moore and Moore [46], the microbial communities that develop on the root surfaces of the teeth that support dentures and draw nourishment from the gingival crevicular fluid are mainly characterized by *Actinomyces naeslundii* in a healthy state.

However, other Actinomyces spp. were also present, such as *Actinomyces meyeri* and *Actinomyces odontolyticus* [47]. *S. sanguinis*, *S. oralis*, *Streptococcus intermedius*, *S. gordonii*, *Peptostreptococcus micros,* and *Gemella morbillorum*, among the Gram-positive species, and *Veillonella parvula*, *Veillonella atypica*, *Capnocytophaga ochracea*, and *Capnocytophaga gingivalis*, among the Gram-negative ones, have also been commonly found in subgingival biofilm. *Fusobacterium nucleatum*, a Gram-negative filamentous, was the second most frequently detected species in healthy subgingival biofilm [46,48].

Subsequent studies have also highlighted the presence of *Rothia aeria*, *Rothia dentocariosa*, *Corynebacterium matruchotti,* and *Corynebacterium durum* [46,47,48,49].

The marginal and internal fit of the prosthetic structure on the tooth is essential for the outcome and maintenance of the fixed prosthetic restoration on natural teeth over time [50]. In case of excessive marginal discrepancy, the cementum layer is thicker, and cementum is the material of the prosthetic structure that is most affected by the oral environment [50]. In fact, cement dissolution, even if minimal, promotes the deposition of dental biofilm, bacterial microleakage, margin discoloration, increased risk of recurrent caries to pulp infection, periodontitis and bone loss, resulting in the risk of prosthetic rehabilitation failure [50].

Although there is no conclusive data in the literature, it appears that computer-aided design (CAD/CAM) allows for increased marginal fit of digitally fabricated restorations [50]. According to many studies zirconia and CAD/CAM lithium disilicate have better marginal fit than hot-pressed lithium disilicate [51]. Neves et al. [52] demonstrated that full-coverage CAD/CAM lithium disilicate crowns offer better horizontal and vertical marginal fit than lost-wax techniques. However, other data show that there is a discrepancy in the closure of crowns obtained by the CAD/CAM method [53]. Porcelain fused metal crowns have greater marginal accuracy to all-ceramic restorations made with CAD/CAM technology [54].

#### 3.3.2. Oral Microbiome in Fixed Dentures on Teeth in Pathological Conditions

Fixed prostheses on teeth have a probability of survival between 89.1–92%, at 10 years [55]. Tooth caries underlying the prosthetic structure are the most common cause of failure [55]. Under certain conditions, an increase in *S. mutans* and *Lactobacilli* can cause demineralization of the tooth which can lead to caries [56].

In dental plaque, normally *S. mutans* serotype c is the most abundant (75%), followed by serotype e (20%), and k (5%) [57]. *S. mutans* has strong cariogenic potential because it is capable of synthesizing, from sucrose, large amounts of extracellular glucan polymers, which help form the extracellular polymeric matrix of the biofilm and colonize hard surfaces of teeth finding fertile ground especially below prosthetic structures with undercuts or due to poor dental hygiene [57]. The low pH environmental conditions, as is the case in the dental biofilm structure, in turn provide a favorable environment for the growth of *S. mutans*, which is also able to transport and metabolize organic carbohydrates further increasing the acidity of the plaque in which it is found [57].

It has been demonstrated by Aas et al. [58] that in the progression of caries the species of the genera *Veillonella*, *Lactobacillus*, *Bifidobacterium*, and *Propionibacterium* can also play a role of fundamental importance.

Periodontal health also plays an important role in the longevity of fixed dental prosthetic restorations [50]. The fine preparation of the prosthetic restoration is the main factor affecting the development of biofilm because high roughness is a favorable environment for the growth of microorganisms [50].

In the case of accumulation of supragingival plaque, gingivitis can occur, i.e., an inflammation of the gingiva characterized by an increase in *Saccharibacteria*, *Leptotrichia*, *Selenomonas*, *Streptococcus*, *Veillonella*, *Prevotella*, *Lautropia,* and *Haemophilus* and by a decrease in *R. dentocariosa*, *Propionibacterium*, and *Stenotrophomonas maltophila* [59,60,61,62,63].

However, more changes at the level of the microbiome are found in the case of periodontitis, which is an irreversible inflammatory disease characterized by the infiltration of immune cells and the destruction of connective tissue, vascular proliferation, and destruction of the alveolar bone [64,65]. In fact, in periodontitis, there is a change in the composition of the subgingival plaque with an increase in the bacteria that make up the so-called triad of the red complex, namely *Porphyromonas gingivalis* (*P. gingivalis*), *Treponema denticola* (*T. denticola*), and *Tannerella forsythia* (*Tib. forsythia*) [60]. There is, however, also an increase in the microorganisms of the orange complex, consisting of *Fusobacterium nucleatum*, *Prevotella intermedia,* and *Parvimonas micra*, as well as *Actinobacillus actinomycetemcomitans*, *Campylobacter rectus*, *Eikenella corrodens*, *Bacteroides forsythus*, *Filifactor alocis*, *Peptoanaerobacter stomatitis*, *Firmicutes phylum*, *Methanobrevibacter oralis*, *Archeon phylotype Thermoplasmata*, *C. Albicans*, Citomegalovirus (CMV), and Epstein-Barr Virus (EBV) [59,60,61,62].

### 3.4. Oral Microbiome in Fixed Dentures on Implants in Healthy and Pathological Conditions

#### 3.4.1. Oral Microbiome in Fixed Dentures on Implants in Healthy Conditions

The main differences between the fixed tooth and implant dentures are due to the presence of the implant screw, the connection system, and the different morphology of peri-implant tissues compared to periodontal tissues.

Similar to the tooth, dental implants exposed in the oral cavity are covered by the acquired film, which consists of an organic layer of proteins, glycoproteins, and lipids [66]. Bacterial colonization begins about 30 min after implant insertion [67] with *Streptococci* and *Actinomyces* setting the stage for colonization of late bacteria such as *Fusobacterium* and *Prevotella* [66]. Early bacterial species can be found on the implant surface as part of the biofilm even several months later [67].

The bacterial characterization of biofilm on implants is similar to the biofilm of nearby natural teeth [66]. The hypothesis is that the oral microbial flora, especially of the neighboring natural teeth, is a “reservoir” for the biofilms of the adjacent implants [66].

Subgingival colonization by oral microorganisms around implants begins approximately 4 weeks after implant placement [66]. The rate of colonization appears to be slower than on natural teeth, probably because of the uncontaminated surface of the implants [66].

The microbiota around dental implants in healthy conditions, according to some authors [66,68], is qualitatively similar to the subgingival microbiota with an equal composition of rods and Gram-positive cocci, followed by nonmotile bacilli and a limited number of Gram-negative anaerobic species [69,70]. However, not all authors agree on this analogy. In fact, some have described substantial differences between periodontal and peri-implant biofilm, potentially attributable to different mechanisms underlying the formation of biofilm on teeth compared to titanium surfaces [71].

The materials most used in dental practice as abutments to be inserted on implants are titanium and zirconium [72,73]. Many studies have compared how bacterial adhesion varies on zirconia discs/abutments compared to titanium discs/abutments [69,74,75].

Investigations on bacterial adhesion to titanium have revealed that corrosion of titanium increases plaque accumulation [72].

Instead, according to in vitro studies, zirconium ceramic frameworks, with or without ceramic coating, are considered promising materials with good biocompatible properties and mechanics [76].

However, in vivo evidence of bacterial adhesion between zirconium and titanium remains controversial. Grossner-Schreiber et al. [69] and Scarano et al. [77] reported that bacterial counts were higher with titanium discs than with zirconia. Instead, Egawa et al. [78] showed that bacterial adhesion to titanium is comparable to that of zirconia if the surfaces of both materials were smooth.

A study performed on 20 edentulous people examined the early colonization of material around zirconia abutments versus titanium abutments prior to final restorations and found no difference in early bacterial colonization between the two materials [73]. Since this observation period was relatively short (3 months) [72,73], further studies are needed to determine whether there are differences in the performance of the structures after final restoration. This is also due to the fact that bacterial colonization could be influenced by the aging of the zirconia [79] and by the long-term corrosion of the titanium [72].

The key factors determining bacterial colonization and adhesion are the roughness of the surface of the material [80,81] and the presence of other dental elements. In fact, the oral flora on the teeth could influence the peri-implant microbiota [75,77]. To eliminate any type of interference, full arch restoration with materials with equal smoothness and no residual teeth should be investigated.

#### 3.4.2. Oral Microbiome in Fixed Dentures on Implants in Pathological Conditions

The design of implant-supported fixed prostheses is the main factor affecting the control of biofilm, which can cause oral and systemic changes, especially in immunocompromised and elderly individuals [82].

Implant-supported prosthetic rehabilitations offer favorable long-term results, but in some cases technical and biological complications may occur, such as peri-implantitis, peri-implant mucositis, prosthetic stomatitis, soft tissue changes, and esthetic and phonetic problems [71,82,83,84].

Biofilm control is difficult especially for full-arch implant-supported fixed prostheses, due to the morphology of the prosthetic support consisting of the fixed abutment in contact with the crestal edentulous mucosa that extends along its entire length [82,85,86].

Because of the favorable environment created for microorganisms at implant retention or prosthetic sites, biofilm accumulation is an important problem for the long-term success of implant rehabilitative treatments [82].

Similar to gingivitis, increased levels of cocci, motile bacilli, and spirochetes occur in cases of peri-implant mucositis [66].

As also happens in the course of periodontitis, in peri-implantitis there is the occurrence of Gram-negative, motile, and anaerobic species [66]. The peri-implantitis microbiome was characterized by high microbial diversity, consisting of aerobic Gram-positive, Gram-negative anaerobic, and pathogenic spindle-shaped rods [66,87]. The characterization of the microbial flora of peri-implantitis-associated biofilms is in concordance with periodontitis, but the presence of *Porphyromonas gingivalis*, *Treponema denticola,* and *Tannerella forsythia* may be greater in peri-implantitis [88]. Several microorganisms that are less commonly found in periodontitis have been identified in peri-implantitis, such as *Staphylococcus aureus*, *Staphylococcus epidermidis*, *Enterobacter aerogenes*, *Enterobacter cloace*, *Escherichia coli*, *Helicobacter pylori*, *Pseudomonas* spp., as well as *Candida* spp. fungi [66]. *P. intermedia*, *C. rectus*, and *Staphylococcus warneri*, as well as *Bacteroidetes* spp., *Actinomyces* spp., *Campylobacter* spp., *Peptococcus* spp., *Streptococcus* spp., and *Butyrivibrio* spp., were also found in peri-implantitis [46].

**Figure 1:**
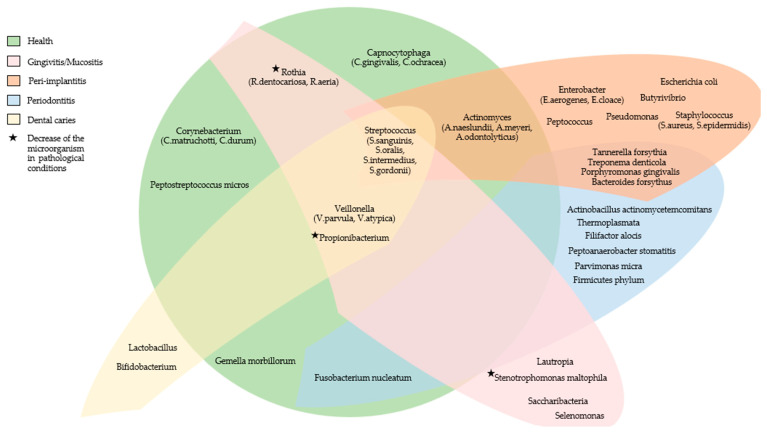
The main microorganisms found in the oral microbiome of subjects with fixed prostheses on teeth or implants in healthy conditions and the main microorganisms that increase in the pathological conditions associated with the dental fixed prosthesis.

Figure 1 summarizes the main microorganisms found in the oral microbiome of subjects with fixed prostheses on teeth or implants in healthy conditions and the main microorganisms that increase in the pathological conditions associated with the dental fixed prosthesis.

### 3.5. Oral Microbiome in Removable Non-Implant-Supported Denture Wearers in Healthy and Pathological Conditions

#### 3.5.1. Oral Microbiome in Removable Non-Implant-Supported Denture Wearers in Healthy Conditions

Another type of prosthetic rehabilitation for individuals with partial or complete edentulism is removable dentures not supported by teeth or implants.

Again, if patients do not maintain proper oral hygiene, local changes may occur in the oral cavity or to the general health of the subject [89].

The prostheses surfaces, usually made of acrylic resin, represent the interface being colonized by oral biofilm a few hours after placement [89]. Under healthy conditions, the microorganisms most commonly present are streptococci, along with Gram-positive and Gram-negative staphylococci, and fungi such as *C. albicans* [89].

The oral mucosa of the cheeks and palate is colonized continuously by microorganisms, despite the continuous exfoliation of the outer layer of the mucosa itself [90], and this makes the colonization of microorganisms on these tissues very impervious [2,3].

The tongue surface, in contrast, has numerous stable niches where microorganisms can settle and create communities. The predominant microbiota on the dorsum of the tongue, characterized by many anaerobic bacteria, in healthy subjects is characterized by *St. salivarius*, *Rothia mucilaginosa*, and an uncharacterized species of *Eubacterium* (FTB41 strain) [91], which are often the cause of bad breath [92].

In particular cases, some microorganisms normally present on the oral mucosa can induce opportunistic infections of the oral cavity or cause systemic diseases in the wearers of partial or total removable dentures. In addition, the presence itself of a dental total prosthesis in the oral cavity causes a decrease in salivary flow and pH, influencing the composition of oral microbiota [43]. Furthermore, in complete dental prostheses, the mucous area covered by the prosthesis does not undergo the cleansing action of the saliva and the cleaning of the tissues by the mechanical action of the tongue, thus favoring the development of biofilm. It should be added that in the elderly the oral tissue loses elasticity, and flakes off more easily, becoming more fragile and susceptible to lesions of the oral cavity, favoring colonization by the opportunistic microbiota [93]. Furthermore, oral hygiene practices by elderly people with cognitive, functional, or neuropsychiatric problems are made much more complicated [94].

#### 3.5.2. Oral Microbiome in Removable Non-Implant-Supported Denture Wearers in Pathological Conditions

Oral mucosal lesions found in removable denture wearers may be acute or chronic reactions to the plaque on the dentures, mechanical lesions induced by the denture, or reactions to the artifact materials (a less frequent condition) [95].

The most commonly found oral lesions are denture stomatitis, angular cheilitis, ulcerative/erosive or hyperplastic traumatic lesions, flaccid ridges, and oral carcinomas [95]. The most common oral condition in partial or total removable denture wearers is denture stomatitis, which is found in about 50% of cases [95,96]. Denture stomatitis is an inflammatory response of the mucosa, usually palatal mucosa, and its clinical manifestation includes chronic edema, erythema, and papillary hyperplasia [96]. Although it has a multifactorial etiology, the main microorganism responsible is *Candida albicans* [95,96]. Baena-Monroy et al. [97] as well as Marchi-Alves [98] concluded that the *Candida albicans*, the *Staphylococcus aureus,* and the *St. mutans* frequently colonize the oral mucosa of denture wearers, especially in cases of prosthetic stomatitis. These pathogens are normal commensals of the oral cavity and do not give particular problems, but they can become pathogenic in the case of immunosuppression drugs or suppression of the host’s defenses [99,100]. The oral mucosa and the supporting tissue of the prosthesis undergo inflammatory alterations, which can become more evident in the event of prolonged use of the prosthetic device. Pathological events are aggravated by unwashed, poorly fitting, broken, or glued prostheses, which damage the patient’s already sensitive mucosa and contribute to the proliferation of fungi and bacteria [101].

Angular cheilitis is a lesion affecting the corners of the mouth inducing maceration, erythema, and crusting [95]. About 15% of total removable denture wearers have angular cheilitis [95]. The microorganism mainly responsible is still *C. albicans* [95].

In contrast, traumatic ulcers induced by the inappropriate prosthesis are found less frequently, in about 5% of cases [95].

Irritative denture hyperplasia is related to chronic lesions on the mucosa in contact with the denture edges and occurs in about 12% of denture wearers [95].

Replacement of the alveolar bone on which the removable denture rests with fibrous tissue leads to the so-called flaccid ridge, which is present in 10–20% of cases [95].

Finally, in rare cases, chronic traumatization of the oral mucosa caused by dentures can lead to the development of carcinomas [95]. The studies of Hooper et al. and Pulshalkar et al. [102,103] have shown that in tumor sites the concentration of *Streptococcus* spp. *oral taxon 058*, *Peptostreptococcus stomatis*, *St. salivarius*, *St. gordonii*, *Gemella haemolysans*, *Gemella morbillorum*, *Johnsonella ignava*, and *Streptococcus parasanguinis* were higher, while *Granulicatella adiacens* was prevalent in non-tumor sites.

Therefore, *Candida albicans* is one of the main microorganisms to cause alterations of the oral mucosa in subjects with removable dentures. In fact, despite the prevalence of bacteria in denture plaque, the most commonly studied organism is the yeast *Candida albicans* [104], and to a lesser extent other Candida species such as *C. glabrata*, *C. famata*, *C. dubliniensis,* and *C. Tropicalis* [105]. *Candida* spp. are well known secondary colonizers of prosthetic plaque in which *C. albicans* can co-aggregate with *Streptococcus* spp. and result in biofilms on saliva-coated surfaces. The yeast is mainly found on the fitting surface of the maxillary prosthesis [13].

Pereira-Cenci’s study [106] evaluated the changes over time in *C. albicans* levels following the insertion of partial or total removable dentures. The study showed that the levels of *C. albicans* decreased after the use of the new prosthesis, but at 6 months they returned to levels similar to the baseline [106]. Furthermore, wearers of complete removable dentures showed higher levels of *Candida albicans* than wearers of removable dentures [106]. Therefore, the study showed that the replacement of old dental prostheses with new prostheses does not allow for a decrease in the excessive rates of *C. albicans* [106].

**Figure 2:**
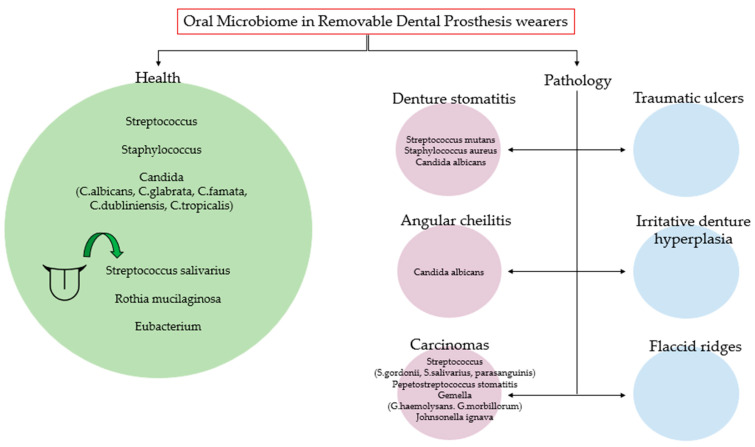
The main microorganisms found in the oral microbiome of subjects with removable prostheses in healthy conditions and the main microorganisms that increase in the pathological conditions associated with the dental removable prosthesis.

Figure 2 summarizes the main microorganisms found in the oral microbiome of subjects with removable prostheses in healthy conditions and the main microorganisms that increase in the pathological conditions associated with the dental removable prosthesis.

### 3.6. Oral Microbiome in Removable Implant-Supported Denture Wearers

The oral microbiota in supported removable denture users has been analyzed by few authors, likely representing a mixture of the microbiome characteristic of fixed implant dentures and removable dentures, also having the major complications found in both prosthetic rehabilitations.

Specifically, a recent cross-sectional study compared the microflora on LOCATOR^®^ attachments supporting removable partial or total overdentures on implants in elderly patients [79]. The pathogenic bacteria were present before the insertion of the prosthesis, but an increase in their concentration was found after 6 months of wearing the prosthesis, despite adequate oral hygiene and proper cleaning of the prostheses [79].

Kilic et al. [107] (2014) evaluated any differences between LOCATOR^®^ and rod overdentures with respect to denture-related stomatitis and Candida species colonization. The authors found more colonies of Candida species in bar overdentures than those retained by LOCATOR^®^. In the same study, the authors found that *C. albicans* was the most common species in bar and LOCATOR^®^ overdentures (81.3% vs. 38.1%, respectively), followed by *Candida glabrata* (37.5% vs. 23.8%, respectively) [107].

### 3.7. Clinical Advice on Cleaning and Disinfection Practices for Dentures

According to the study by Ryniewicz et al. [89], conducted on 120 subjects divided into 2 groups (group I: subjects with fixed restorations; group II: subjects with removable restorations), the oral hygiene of denture wearers is inadequate and does not depend on the type of denture.

This result emphasizes the importance of educating fixed or removable denture wearers on maintaining good oral hygiene.

#### 3.7.1. Clinical Advice on Cleaning and Disinfection Practices for Implant-Supported Prostheses

Plaque removal on implant-supported prostheses is an important long-term prognostic factor for maintenance and stability of dental implants and for the prevention of biological complications [108].

The 2017 World Workshop Consensus report on periodontal and peri-implant conditions defined peri-implant mucosa as healthy when there is not erythema, bleeding on probing, swelling, or suppuration, while defining peri-implant mucositis as the presence of clinical inflammation, and peri-implantitis as the additional presence of progressive bone loss [108].

Several authors have in fact demonstrated that peri-implantitis causing the failure of dental implants has been associated with higher levels of plaque biofilm compared to normally osseointegrated implants.

A recent study confirmed the association between peri-implantitis and incorrect bone design implant-supported prostheses [109].

Of fundamental importance is the hygienic maintenance of prostheses supported by fixed implants and above all the conformation of the prosthesis itself. In fact, a recent study described how peri-implantitis mostly occurred in implant sites where there was a lack of access for daily hygiene practices and therefore inadequate plaque control [109].

Different methods for the home control of plaque on implant-supported prostheses have been described in the literature; these include the use of manual or electric toothbrushes and dental devices for proximal cleaning [110].

A recent systematic review investigated the best home oral hygiene methods for implant-supported restorations [110]. It has been found that electric toothbrushes perform better and lead to an improvement in clinical parameters over time compared to manual toothbrushes together with water jet devices [110].

Furthermore, other controlled clinical studies have found that electric toothbrushes gave better results than manual toothbrushes in subjects rehabilitated with fixed prostheses [111,112].

However, a recent controlled clinical study has shown comparable efficacy between the manual and electric toothbrush in edentulous elderly subjects with implant-supported overdentures [113].

Finally, Tawse-Smith et al. [113] conducted a study for only a short period of time (6 weeks), which could lead to a bias in the results. There is a paucity of studies investigating interproximal devices [110]. The oral hygiene measures administered by professionals have been suggested to play a major role in prevention [114,115]. A study conducted in Spain showed that adequate maintenance and recall of oral hygiene, twice a year, was associated with 86% fewer complications than those who did not adhere to this protocol [116].

The use of home adjuvant products, such as ozonated gels, against different species of candida and the use of probiotics and postbiotics to reduce the incidence of oral dysbiosis and to improve the healing process is proposed by Srcibante groups. All these professional and home-based approaches can improve the quality of patients’ home hygiene methods [117,118,119].

Smoking should also be kept under control, as it is considered another important risk factor for the change in the oral microbiome and for the influence on periodontal and peri-implant parameters [120,121].

#### 3.7.2. Clinical Advice on Cleaning and Disinfection Practices for Removable Prosthesis

The hygiene of removable prosthesis is one of the most important factors for wearers of removable dentures. The main objective of all oral hygiene interventions for wearers of removable prostheses is to eliminate the pathogenic microorganisms which can colonize the surfaces of the prosthesis itself [122,123]. Therefore, it becomes important to keep the pathogenic biofilm under control that could develop on the prosthesis.

Biofilm could then invade other districts of the oral cavity [4], even causing serious infections in patients who are immunosuppressed or have a compromised immune system [124].

The removal of the biofilm from the prosthesis can be obtained in various ways including the application of hygienic methods through mechanical methods, chemical agents and irradiation, or a combination of the above methods [123,124].

The most commonly used mechanical method is denture brushing, which is the simplest and cheapest method [125].

As far as chemical cleaning methods are concerned, there is a wide range of treatments, including the use of hypochlorite, peroxides, enzymes, acids, and mouthwashes. These methods can, however, also be used in combination with each other [125].

The method of biofilm removal through the irradiation of dentures involves the use of photodynamic therapy (PDT) or the application of microwaves and has also been described as an effective method for disinfecting full dentures [125,126].

An ideal method of denture hygiene should, in addition to the bactericidal and fungicidal action, ensure that the physical–mechanical properties of both the prosthetic base and the prosthetic teeth remain unchanged. In particular, the color, size, and stability of the material used for the prostheses are important prerequisites for maintaining and preserving the longevity of the prosthesis over time [123].

A recent review [126] has highlighted how physical action, such as brushing or ultrasonic vibration in association with chemical agents, leads to more effective results in reducing plaque and the number of microorganisms present on the prosthesis, compared with other single methods. The dimensional stability seems to be unchanged; however, few studies have investigated this variation. Too high concentrations of disinfectant solutions can in fact modify the color stability of the prosthesis.

Brushing, however, remains the most common hygienic practice for cleaning prostheses [126].

## 4. Conclusions

Both fixed and removable dental prostheses, on teeth and on implants, are subject to bacterial colonization and can contribute to the formation of bacterial plaque.

To avoid the accumulation of large quantities of plaque and pathogenic bacteria, it is important to carry out daily hygiene procedures for teeth, implant-supported crowns, fixed bridges, and removable prosthetic devices, paying close attention to elderly patients who may not be able to perform the correct oral hygiene practices, and use products against plaque accumulation or oral dysbiosis, as in the case of chlorhexidine, ozone-based products, probiotics and postbiotics, to improve patients’ home oral practices [117,118].

It is important to educate patients and advise them to maintain minimal levels of plaque through regular checkups by their dentist.

Hygiene is also important for removable partial prostheses which must be adequately cleaned and disinfected with special disinfectant products.

Particular attention is needed for periodontal patients, as the periodontal or peri-implant pockets could act as a reservoir for bacteria, viruses, and fungi which could subsequently colonize other parts of the oral cavity or the prosthetic products.

However, it is also very important to design the prosthesis well, especially if supported by implants and fixed, to facilitate home oral hygiene practices by the patient and avoid peri-implantitis and the consequent loss of dental implants, which would compromise the stability of the prosthesis itself.

Further studies are needed to compare the different materials used in prostheses to better understand which materials are more biocompatible and which render bacterial adhesion more difficult.
